# Technical and Clinical Outcome of Talent versus Endurant Endografts for Endovascular Aortic Aneurysm Repair

**DOI:** 10.1371/journal.pone.0038468

**Published:** 2012-06-08

**Authors:** Birger Mensel, Jens-Peter Kühn, Tobias Träger, Martin Dührkoop, Wolfram v. Bernstorff, Christian Rosenberg, Andreas Hoene, Ralf Puls

**Affiliations:** 1 Institute of Diagnostic Radiology and Neuroradiology, University Medicine Greifswald, Greifswald, Mecklenburg-Vorpommern, Germany; 2 Department of Vascular Surgery, University Medicine Greifswald, Greifswald, Mecklenburg-Vorpommern, Germany; Policlinico San donato milanese, Italy

## Abstract

**Objective:**

The technical evolution of endografts for the interventional management of infrarenal abdominal aortic aneurysms (AAA) has allowed a continuous expansion of indications. This study compares the established Talent endograft with its successor, the Endurant endograft, taking individual aortoiliac anatomy into account.

**Methods:**

From June 2007 to December 2010, 35 patients with AAA were treated with a Talent endograft (33 men) and 36 patients with an Endurant endograft (34 men). Aortoiliac anatomy was evaluated in detail using preinterventional computed tomography angiography. The 30-day outcome of both groups were compared regarding technical and clinical success as well as complications including endoleaks.

**Results:**

The Endurant group included more patients with unfavorable anatomy (kinking of pelvic arteries, p = 0.017; shorter proximal neck, p = 0.084). Primary technical success was 91.4% in the Talent group and 100% in the Endurant group (p = 0.115). Type 1 endoleaks occurred in 5.7% of patients in the Talent group and in 2.8% of those in the Endurant group (p = 0.614). Type 3 endoleaks only occurred in the Talent group (2.9% of patients; p = 0.493). Type 2 endoleaks were significantly less common in the Endurant group than in the Talent group (8.3% versus 28.6%; p = 0.035). Rates of major and minor complications were not significantly different between both groups. Primary clinical success was significantly better in the Endurant group (97.2%) than in the Talent group (80.0%) (p = 0.028).

**Conclusion:**

Endurant endografts appear to have better technical and clinical outcome in patients with difficult aortoiliac anatomy, significantly reducing the occurrence of type 2 endoleaks.

## Introduction

Endovascular aneurysm repair (EVAR) has become the method of choice for treating infrarenal abdominal aortic aneurysms (AAA). This is largely due to its minimal invasiveness and the continuously improved outcome with low morbidity and mortality [1], [2]. In addition to aortoiliac anatomy, the material of which a stent-graft is made and the endograft design are other important factors contributing to outcome after EVAR [3], [4], [5], [6]. A great variety of different models and manufacturers are currently available. They differ in basic design, the endograft material used, the site of proximal fixation, and the presence of anchoring hooks or pins at the proximal graft end.

The development of new endografts aims at effectively and permanently reducing pressure within the aneurysm sac. Thereby, reinterventions due to a persistent or recurrent perfusion of the aneurysm are prevented. Moreover, improved stent-grafts can also be used for EVAR in patients with difficult aortoiliac anatomies such as short proximal necks, severely angulated infrarenal aortas, and kinking or heavy calcification of pelvic arteries [4], [7], [8].

A fairly new model of endografts is the Endurant endograft, which evolved from its predecessor, the Talent endograft (both manufactured by Medtronic Vascular, Santa Rosa, USA). Optimizing the physical and mechanical properties of both, the endograft itself and the delivery system has further increased the range of indications compared with its predecessor [7], [9], [10]. This has been accomplished by forming sinusoidal M-shaped main body stents for greater radial force and flexibility as well as reducing the diameter of the hydrophilic coated delivery system.

In the present study interventional and postinterventional outcomes of the Talent and Endurant stent-grafts were compared. A specific parameter of interest was the individual anatomy of the aortoiliac vascular system of the patients treated.

## Materials and Methods

### Patients

The institutional review board (the Ethics Committee of the University of Greifswald) approved this retrospective study (Approval No. BB 128/11), which complied with the principles of the Declaration of Helsinki (2008 version). All patients gave written informed consent to participate.

From June 2007 to December 2010, 72 patients with AAA were treated with an infrarenal Talent or Endurant endograft. Inclusion criteria for this retrospective study were elective or emergency endovascular repair of an AAA using a Talent or Endurant stent-graft. Patients were excluded if their preinterventional and postinterventional files (at least up to 30 days after the intervention) were incomplete or if adequate pre-or postinterventional computed tomography angiography (CTA) or digital subtraction angiography datasets were not available. One of the 72 consecutive patients was excluded because no adequate postinterventional CTA was available.

Hence, 71 patients were finally included in the study, 35 in the Talent group (49.3%) and 36 (50.7%) in the Endurant group. Sixty-seven patients were male (94.4%), four were female (5.6%). The mean age of all patients was 76±7 years. Basic data did not differ significantly between the two groups. The data are summarized in Table 1.

**Table 1:** Basic data of study patients.

Patient data	Talent N = 35 Mean orN (range or %)	Endurant N = 36 Mean orN (range or %)	p
Age [years]	75±8 (58–91)	78±6 (68–90)	0.109
Men	33 (94.3)	34 (94.4)	1.000
Hypertension	27 (77.1)	30 (83.3)	0.721
Obesity	6 (17.1)	4 (11.1)	0.514
Hyperlipidemia	15 (42.9)	19 (52.8)	0.549
Smoking	26 (74.3)	27 (75.0)	0.877
Renal insufficiency	8 (22.9)	6 (16.7)	0.721
PAOD	14 (40.0)	17 (47.2)	0.708

*PAOD* Peripheral artery occlusive disease.

### Preinterventional Diagnostic Evaluation

All patients underwent contrast-enhanced CTA before intervention (LightSpeed, 8 rows, GE Healthcare, Munich, Germany). CTA was performed with 5-mm slice thickness, a pitch of 1.35∶1, and 1.25-mm collimation. The contrast agent (Imeron 350, BRACCO Imaging, Constance, Germany) was administered intravenously at a dose of 100–120 ml and a flow of 4 ml/s using bolus tracking. The standard procedure included subsequent axial reconstruction at 1.25-mm slice thickness, followed by coronal and sagittal reformation. These images were used to plan EVAR, which was performed within two weeks of CTA. The reconstructed axial image series were analyzed using the OsiriX image viewer (version 3.9.2, Pixmeo Sarl, Bernex, Switzerland). The following parameters were determined to assess the aortoiliac anatomy:

1. Length and maximum diameter of the proximal neck.
2. Length and maximum diameter (including mural thrombus) of the aneurysm sac.
3. Angles between the suprarenal aortic axis and the axis of the proximal neck (suprarenal angle) and between the proximal neck and body of the aneurysm (infrarenal angle).
4. Angle between the axes of the common iliac arteries (bifurcation angle).
5. Tortuosity index expressed as the ratio of the actual length of the common iliac artery down to the mid-common femoral artery (puncture site) over the direct distance from end to end.
6. Evaluation for kinking of the common/external iliac artery (right-or acute-angled course of the vessel) and aneurysm extension to at least one common iliac artery.
7. Smallest arterial diameter at the site of ilio-femoral access.

All angles and distances were measured orthogonal to or along the vascular axis, using double oblique multiplanar reformations when needed. Vascular diameters were measured from inner wall to inner wall (perfused lumen).

### Choice of Stent-Graft and Intervention

All interventions were performed under aseptic conditions using the same angiography system (Axiom-Artis, Siemens, Erlangen, Germany) by a multidisciplinary team including interventional radiologists and vascular surgeons, each having at least 5 years of experience in endovascular treatment of AAA. Talent endografts were used until February 2009, Endurant grafts thereafter.

The contrast agent used was Imeron 300 (BRACCO Imaging, Constance, Germany). All interventions were performed under general anesthesia, using bilateral open inguinal surgical access to the common femoral artery according with the manufacturer’s instructions. Most electively treated patients received bifurcated endografts, while aortomonoiliac endografts were used in all patients undergoing emergency treatment for AAA. In addition, a crossover bypass graft was carried out in these patients during the same session. Following EVAR all patients were transferred to the intensive care unit.

### Follow-up

The follow-up period was 30 days. Before discharge from the hospital, all patients underwent clinical examinations, laboratory tests (creatinine, urea, hemoglobin, blood count), and CTA (same CT scanner and technical parameters as for the preinterventional examinations). In addition, a venous phase series was acquired 45–60 sec after contrast medium injection. Postinterventional CTA was performed within 3–30 days of endograft implantation.

### Definitions

The definitions below are in accordance with the recommendations of the Ad Hoc Committee for Standardized Reporting Practices in Vascular Surgery of the Society for Vascular Surgery [11].

Primary technical success required the successful introduction and deployment of the device in the absence of surgical conversion or mortality, type I or III endoleaks, or graft limb obstruction without an unplanned endovascular or surgical procedure.

Assisted primary technical success was defined as the need for additional endovascular or surgical procedures to achieve the above-mentioned aims.

Primary clinical success required successful deployment of the endovascular device at the intended location without death as a result of aneurysm-related treatment, type I or III endoleak, graft infection or thrombosis, aneurysm expansion or rupture, or conversion to open repair without the need for an additional or secondary surgical or endovascular procedure.

Assisted primary clinical success was to achieve the above-mentioned goals with the use of an additional or secondary endovascular procedure.

The classification of endoleaks is summarized in Table 2.

**Table 2:** Classification of endoleaks*.

Type of endoleak	Cause of perigraft flow
**I**	a) Inadequate seal at proximal end of endograft b) Inadequate seal at distal end of endograft c) Inadequate seal at iliac occluder plug
**II**	Flow from visceral vessel (lumbar, mesenteric inferior, hypogastric artery) a) Single vessel (simple) b) At least two vessels creating a circuit (complex)
**III**	Flow from module disconnection
**IV**	Flow from porous fabric (<30 days after graft placement)

* modified according to [11].

Complications were categorized as minor or major. Minor complications were all undesired events that not required surgical treatment, recovered spontaneously or within 24 hours (e.g. hematoma at access site, pneumonia treated with oral antibiotics). Major complications were defined as those that required an invasive treatment or led to hospitalisation >24 h (e.g. limb occlusion treated by surgical intervention).

### Statistical Analysis

Statistical analysis was performed using MedCalc (version 11.5.1.0, Mariakerke, Belgium).

Quantitative measurements were expressed as mean ± standard deviation. Categorical data were tested using the χ^2^ test or Fisher’s exact test. Continuous data were analyzed with the Mann-Whitney U-test. Statistical significance was assumed at p<0.05.

## Results

### Aortoiliac Anatomy

The proximal neck had a mean length of 3.96±0.19 cm in the Talent group and was on average 0.75 cm shorter in the Endurant group (3.21±1.35 cm, p = 0.084). The suprarenal and infrarenal angles were not significantly different between the two groups (p = 0.828 and 0.836) (Table 3). Kinking of the common/external iliac artery was present in 41.7% (15/36) of patients in the Endurant group, which was significantly more common than in the Talent group with 14.3% (5/35; p = 0.017). The maximum aneurysm diameter was significantly smaller in the Talent group compared with the Endurant group (5.28±1.50 cm versus 5.89±1.59; p = 0.037).

**Table 3:** Morphologic criteria for evaluating aortoiliac anatomy.

Morphologic criteria	Talent N = 35 Mean orN (range or %)	Endurant N = 36 Mean orN (range or %)	p
Length of proximal neck [cm]proximal neck <1.5 cm	3.96±0.19 (0.92–6.91) 5 (14.3%)	3.21±1.35 (0.45–5.86) 5 (13.9)	0.084 1.000
Diameter of proximal neck [cm]	2.47±0.32 (1.86–3.33)	2.50±0.42 (1.88–3.59)	0.902
Suprerenal angle [°]	13.6±12.6 (1.0–58.2)	14.6±14.5 (2.1–57.6)	0.828
Infrarenal angle [°]	33.0±15.3 (1.3–73.2)	37.6±17.3 (7.4–86.2)	0.836
Maximum diameter of aneurysm sac [cm]	5.28±1.50 (3.21–9.45)	5.89±1.59 (2.65–10.68)	0.037
Length of aneurysm sac [cm]	7.71±2.94 (1.14–14.8)	8.03±3.46 (2.67–16.8)	0.818
Bifurcation angle [°]	48.6±24.5 (12.3–120.5)	52.1±28.6 (4.5–122.2)	0.633
Aneurysm extension to common iliac artery	6 (17.1)	4 (11.1)	0.514
Kinking of common/external iliac artery	5 (14.3)	15 (41.7)	0.017
Tortuosity index, right	1.28±0.24 (1.00–1.96)	1.22±0.21 (1.00–1.86)	0.373
Tortuosity index, left	1.26±0.26 (1.04–2.24)	1.26±0.27 (1.00–2.25)	0.713
Tortuosity index, side from which main endograft body was introduced	1.27±0.23 (1.00–1.96)	1.26±0.22 (1.00–1.86)	0.486
Minimum diameter at access site, right [cm]	0.61±0.14 (0.33–0.92)	0.67±0.20 (0.23–1.36)	0.119
Minimum diameter at access site, left [cm]	0.65±0.14 (0.39–0.93)	0.67±0.19 (0.35–1.19)	0.904
Minimum diameter, side from which main endograft body wasintroduced [cm]	0.62±0.14 (0.39–0.93)	0.67±0.21 (0.23–1.36)	0.294

### Intervention-related Data

The interventions were elective in 91.4% (32/35) of the patients in the Talent group and in 91.7% (33/36) of the patients in the Endurant group; three patients in each group were treated for retroperitoneally ruptured AAA (p = 1.000).

In the Talent group, the stent-graft system could be introduced and the graft deployed as planned in 97.1% (34/35) of the patients. In one patient, the guidewire perforated the external iliac artery, requiring an iliacofemoral bypass through which the main stent-graft body could be introduced.

In the Endurant group, the endograft was delivered as planned in 100.0% (36/36) of the patients (p = 1.000).

The duration of the procedure (biiliac stent-grafts) was 124.0±16.7 min in the Talent group and 115±24.7 min in the Endurant group; the difference was not statistically significant (p = 0.146).

The primary technical success rate was 91.4% (32/35) in the Talent group. Reasons for technical failure were perforation of the external iliac artery in the patient already mentioned. In one patient a small type 1a endoleak was detected immediately after the intervention (proximal neck length: 0.12 cm) and persisted despite repeated balloon dilatation. However, no reintervention was performed within the follow-up period. One patient had a dissection of the external iliac artery occurring during introduction of the main stent-graft body. In this patient, subsequent percutaneous transluminal angioplasty (PTA) with stent implantation in the same session resulted in assisted primary technical success (Table 4).

**Table 4:** Intervention-related data of the Talent and Endurant groups.

Intervention-related data	Talent N = 35 Mean orN (range or %)	Endurant N = 36 Mean orN (range or %)	p
Elective intervention	32 (91.4)	33 (91.7)	1.000
Biiliac endograft	28 (80.0%)	33 (91.7%)	0.189
Duration of intervention, biiliac graft [min]	124.0±16.7 (90.0–164.0)	115.0±24.7 (41.0–162.0)	0.146
Duration of intervention, monoiliac graft [min]	131.0±34.8 (74.0–168.0)	116.0±47.8 (32.0–169.0)	0.689
Primary technical success	32 (91.4)	36 (100.0)	0.115
Assisted primary technical success	33 (94.3)	36 (100.0)	0.239
Primary endoleak			
type 1	2 (5.7)	1 (2.8)	0.614
type 2	10 (28.6)	3 (8.3)	0.035
type 3	1 (2.9)	0	0.493

In the Endurant group, primary technical success was achieved in 100% (36/36; p = 0.115).

### Endoleaks

Type 1 primary endoleaks occurred in 5.7% (2/35) of the patients of the Talent group. As already mentionezd, one type 1a endoleak occured during the implantation procedure. Another type 1a endoleak was detected at follow-up and was successfully treated by proximal stent-graft extension. Further follow-up revealed a patient with a type 3a endoleak (at the intermodular connection site of the contralateral limb and the main body), which was treated by implantation of another covered stent-graft.

In the Endurant group, follow-up detected one type 1a endoleak (2.8%) which could be adequately treated by embolization using coils and histoacryl.

**Figure 1:**
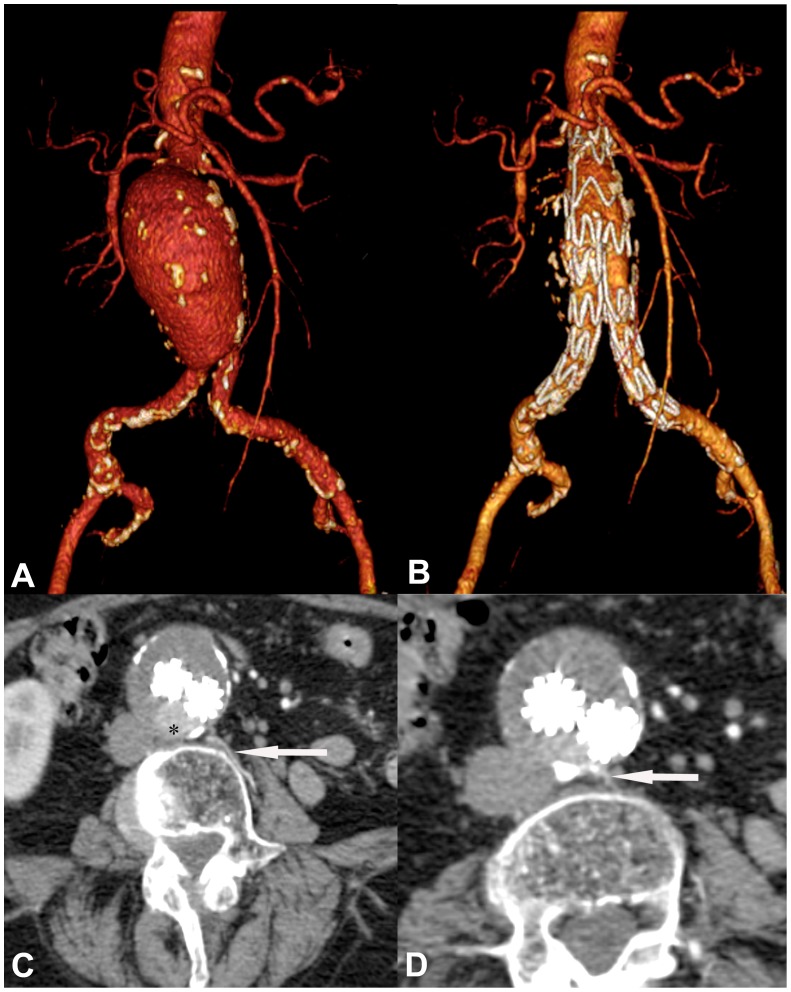
CTA of a large infrarenal AAA in a 79-year-old patient. **A,** Volume reconstruction (VR) of CTA with moderate calcification within the aneurysm sac and both common iliac arteries. **B,** VR performed 12 days after implantation of a Talent stent graft. **C,** Venous phase CTA reveals a type 2 endoleak (asterisk) posterior of the stent graft limb and a perfused lumbar artery on the left (arrow). **D,** More inferiorly, the site of entry of the lumbar artery into the aneurysm sac is seen (arrow).

**Figure 2:**
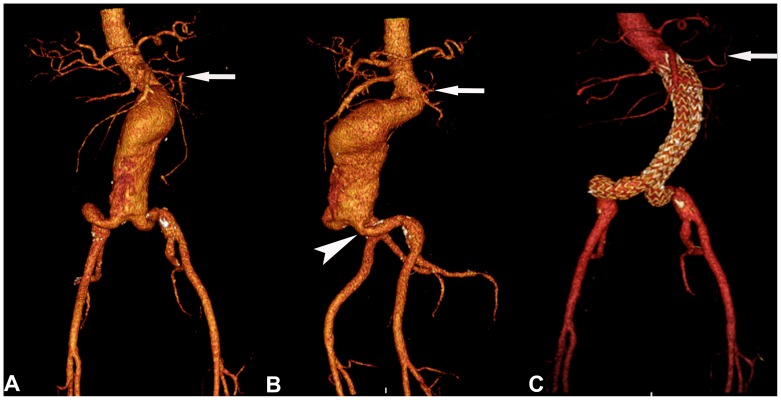
CTA of an infrarenal AAA in a 74-year-old patient. A, VR of the long aneurysm with a short proximal neck. The aneurysm involves the aortic bifurcation, and there is marked angulation of the infrarenal portion (arrow indicates the left renal artery). Both common iliac arteries are markedly elongated. **B,** Lateral VR more clearly showing the elongation of the left common iliac artery and also severe kinking (arrowhead) at its origin as well as marked infrarenal angulation of the proximal neck (arrow indicates the left renal artery). **C,** Postinterventional VR indicating successful implantation of an Endurant stent graft and exclusion of AAA (arrow indicates the left renal artery).

In the Talent group, 28.6% of the patients (10/35) had type 2 endoleaks, including 8 type 2a endoleaks and 2 type 2b endoleaks (Fig. 1). One type 2a endoleak occurred in a patient who also had a type 1 endoleak. In the Endurant group, only 8.3% (3/36) type 2 endoleaks (all type 2a) occured. This was significantly less than in the Talent group (p = 0.035) (Fig. 2). So far, no reinterventions were required for type 2 endoleaks in either group.

### Other Major and Minor Complications

No patient died within 30 days of the intervention. In the Talent group, there was one graft limb occlusion (5th postinterventional day). The patient was successfully treated by open surgical thrombectomy. A second patient with stenosis of the iliac limb (detected 7 days after the intervention) successfully underwent PTA with stenting. In the Endurant group, one patient had a myocardial infarction and required intensive care treatment, which markedly prolonged the hospital stay. Mesenteric ischemia, stroke, or heavy blood loss due to the intervention (>1000 ml) did not occur in any patient. The major complication rate was 2.9% (1/35) in the Talent group and 2.8% (1/36) in the Endurant group; the difference was not significant (p = 1.000) (Table 5). The minor complication rate was 5.7% (2/35) and 11.1% (4/36) respectively; again the difference was not statistically significant (p = 0.647).

**Table 5:** Outcome at 30-day follow-up in the Talent versus Endurant group.

30-day outcome	Talent N = 35 N (%)	Endurant N = 36 N (%)	p
Mortality	0	0	1.000
Major complications			
Occlusion of iliac limb	1 (2.9)*	0	0.493
Myocardial infarction	0	1 (2.8)*	1.000
*Total*	1 (2.9)	1 (2.8)	1.000
Minor complications			
Hematoma at access site	1 (2.9)	2 (5.6)	1.000
Stenosis of iliac limb	1 (2.9)	0	0.493
Renal infarction	0	1 (2.8)^ †^	1.000
Pneumonia	0	1 (2.8)	1.000
*Total*	2 (5.7)	4 (11.1)	0.674
Endovascular reintervention	3 (8.6)	1 (2.8)	0.357
Primary clinical success	28 (80.0)	35 (97.2)	0.028
Assisted primary clinical success	32 (91.4)	35 (97.2)	0.357

* Classified as clinical failure. ^†^ Small embolic renal infarction at lower pole after the intervention without clinically relevant impairment of renal function.

Endovascular reinterventions were performed in 8.9% (3/35) of the patients of the Talent group and in 2.8% (1/36) of the patients of the Endurant group (p = 0.375).

The median intensive care unit stay was 1 day (range, 0–32) for all patients. The median total hospital stay was 6 days (range, 2–36).

The primary clinical success rate after 30 days was 80.0% (28/35) in the Talent group and 97.2% (35/36) in the Endurant group (p = 0.028).

## Discussion

The continuous improvement of available endografts and delivery systems has increased the indications for EVAR in patients with AAA [7], [12], [13]. The broader spectrum of indications using state-of-the-art stent-grafts is particularly beneficial for patients with difficult aortoiliac anatomy. This includes short proximal necks (<1 cm), severe supra- and infrarenal aortic angulation or elongation and kinking of pelvic arteries [6], [7], [9]. To better fit these anatomical variants the Endurant endograft has been developed.

In this study, we compared the established Talent endograft with its successor, the Endurant endograft.

Comparison of baseline data shows that more patients with difficult anatomy have been treated using an Endurant endograft. Kinking of the common/external iliac artery was significantly more common in the Endurant group (41.7 versus 14.3%; p = 0.017). The proximal neck was on average 0.75 cm shorter in the Endurant group, but the difference falls short of statistical significance (p = 0.084). The primary technical success rate was slightly lower in the Talent group compared with the Endurant group (91.4% versus 100%; p = 0.115). These results are in agreement with data reported in the literature with rates of 91–99% for Talent endografts and 98–100% for Endurant endografts [7], [9], [14], [15], [16]. Another incentive for designing new stent-grafts is to minimize the need for secondary interventions to maintain graft function. Endoleaks, stent dislocation and limb occlusion are the three most important reasons for reinterventions. A leak can result in reperfusion of the aneurysm sac and is associated with an increased risk of aneurysm rupture [15], [16], [17], [18]. In this study, the rates of primary type 1 endoleaks were not significantly different between the Talent (5.7%) and Endurant (2.8%) group (p = 0.614). A trend towards slightly higher incidences of type 1 endoleaks for Talent versus Endurant endografts has also been reported in the literature (8–12% versus 0–6%, depending on the underlying aneurysm anatomy) [4], [7], [15], [19]. The better results for Endurant endografts might be attributable to the changed design of the proximal end of the suprarenal stent, which has an additional set of 6 paired anchor pins. Also, additional M stents are now provided that have been designed to improve proximal anchorage and sealing.

Type 3 endoleaks were only observed in one patient of the Talent group (2.9%, p = 0.493); this type of endoleak is rare with both models of endografts [4], [15]. In contrast, type 2 endoleaks are much more common with their incidence ranging from 6% to 30%, depending on the type of endograft used [2], [3], [7]. In the present study, type 2 endoleaks were significantly more common in the Talent group (28.6% versus 8.3% in the Endurant group; p = 0.035). The occurrence of type 2 endoleaks with different endograft systems was also investigated by Sheehan et al., who also found only slight differences between the systems [3]. They attributed the differences to different endograft designs including their different mechanical properties. The more flexible structure of Endurant endografts might improve their alignment to the aortic wall and/or the mural thrombus, sealing the remaining arterial branches e.g. lumbar arteries or the inferior mesenteric artery, which may otherwise relevantly perfuse the aneurysm sac. Thus, these branches might be occluded by the endograft, preventing a type 2 endoleak. Although type 2 endoleak is still quite common, there is an ongoing controversy regarding its clinical relevance with regard to aneurysm growth or rupture including the need for reinterventions and their best timing. Treatment of persisting type 2 endoleaks is mandatory because prolonged blood inflow into the aneurysm sac will significantly increase pressure within the aneurysm sac, which may reach levels of up to 70–80% of systemic blood pressure [20], [21], [22]. In this study population, there have not been any reinterventions for type 2 endoleaks so far. However, this may be due to the short follow-up period. Another limitation is the small number of patients included, which is due to the single-center design. Other limitations include the lack of randomization, the retrospective design as well as the inclusion of both elective and emergency procedures. Moreover, a learning curve has to be taken into account, as the two endograft systems were used successively, and the interventionalists’ earlier experience with the Talent endograft has probably improved their handling of the Endurant grafts and may have reduced complications.

Major and minor complications did not differ significantly between the two groups (p = 1.000 and 0.647, respectively). No compression of the iliac limb with subsequent thrombosis was observed, which was a common complication described after Endurant procedures by Makaroun et al. [4]. As a possible cause the authors discussed the greater flexibility of the Endurant graft, which, while improving navigation, increases the risk of collapse of the stent-graft lumen. Yet, in the present study the Endurant group showed a significantly better result in terms of primary clinical success (97.2% versus 80.0% for Talent; p = 0.028).

To avoid the above limitations and to confirm these results randomized multicenter studies in larger patient populations including their long-term follow-up are required.

In conclusion, this results suggests that Endurant endografts have a better outcome with a significantly lower rate of type 2 endoleaks compared to Talent endografts despite a higher proportion of patients with difficult aortoiliac anatomy in the Endurant group.

## References

[1] Greenhalgh RM, Brown LC, Kwong GP, Powell JT, Thompson SG (2004). Comparison of endovascular aneurysm repair with open repair in patients with abdominal aortic aneurysm (EVAR trial 1), 30-day operative mortality results: randomised controlled trial.. Lancet.

[2] Turnbull IC, Criado FJ, Sanchez L, Sadek M, Malik R (2010). Five-year results for the Talent enhanced Low Profile System abdominal stent graft pivotal trial including early and long-term safety and efficacy.. Journal of vascular surgery: official publication, the Society for Vascular Surgery and International Society for Cardiovascular Surgery, North American Chapter.

[3] Sheehan MK, Ouriel K, Greenberg R, McCann R, Murphy M (2006). Are type II endoleaks after endovascular aneurysm repair endograft dependent?. Journal of vascular surgery : official publication, the Society for Vascular Surgery [and] International Society for Cardiovascular Surgery, North American Chapter.

[4] Makaroun MS, Tuchek M, Massop D, Henretta J, Rhee R (2011). One year outcomes of the United States regulatory trial of the Endurant Stent Graft System.. Journal of vascular surgery: official publication, the Society for Vascular Surgery [and] International Society for Cardiovascular Surgery, North American Chapter.

[5] Ohrlander T, Dencker M, Acosta S (2011). Morphological State as a Predictor for Reintervention and Mortality After EVAR for AAA..

[6] Choke E, Munneke G, Morgan R, Belli AM, Loftus I (2006). Outcomes of endovascular abdominal aortic aneurysm repair in patients with hostile neck anatomy.. Cardiovascular and interventional radiology.

[7] Hyhlik-Durr A, Weber TF, Kotelis D, Rengier F, Gahlen J (2011). The Endurant Stent Graft System: 15-month follow-up report in patients with challenging abdominal aortic anatomies.. Langenbeck’s archives of surgery.

[8] Troisi N, Torsello G, Donas KP, Austermann M (2010). Endurant stent-graft: a 2-year, single-center experience with a new commercially available device for the treatment of abdominal aortic aneurysms.. Journal of endovascular therapy: an official journal of the International Society of Endovascular Specialists.

[9] van Keulen JW, de Vries JP, Dekker H, Goncalves FB, Moll FL (2011). One-year multicenter results of 100 abdominal aortic aneurysm patients treated with the Endurant stent graft.. Journal of vascular surgery: official publication, the Society for Vascular Surgery and International Society for Cardiovascular Surgery, North American Chapter.

[10] Rouwet EV, Torsello G, de Vries JP, Cuypers P, van Herwaarden JA (2011). Final results of the prospective European trial of the Endurant stent graft for endovascular abdominal aortic aneurysm repair.. European journal of vascular and endovascular surgery: the official journal of the European Society for Vascular Surgery.

[11] Chaikof EL, Blankensteijn JD, Harris PL, White GH, Zarins CK (2002). Reporting standards for endovascular aortic aneurysm repair.. Journal of vascular surgery: official publication, the Society for Vascular Surgery and International Society for Cardiovascular Surgery, North American Chapter.

[12] Broker HS, Foteh KI, Murphy EH, Davis CM, Clagett GP (2008). Device-specific aneurysm sac morphology after endovascular aneurysm repair: evaluation of contemporary graft materials.. Journal of vascular surgery: official publication, the Society for Vascular Surgery [and] International Society for Cardiovascular Surgery, North American Chapter.

[13] Murphy EH, Arko FR (2008). Technical tips for abdominal aortic endografting.. Seminars in vascular surgery.

[14] Georgiadis GS, Trellopoulos G, Antoniou GA, Gallis K, Nikolopoulos ES (2011). Early results of the Endurant endograft system in patients with friendly and hostile infrarenal abdominal aortic aneurysm anatomy.. Journal of vascular surgery: official publication, the Society for Vascular Surgery and International Society for Cardiovascular Surgery, North American Chapter.

[15] Verhoeven BA, Waasdorp EJ, Gorrepati ML, van Herwaarden JA, Vos JA (2011). Long-term results of Talent endografts for endovascular abdominal aortic aneurysm repair.. Journal of vascular surgery: official publication, the Society for Vascular Surgery [and] International Society for Cardiovascular Surgery, North American Chapter.

[16] Pitton MB, Scheschkowski T, Ring M, Herber S, Oberholzer K (2009). Ten-year follow-up of endovascular aneurysm treatment with Talent stent-grafts.. Cardiovascular and interventional radiology.

[17] Coppi G, Silingardi R, Saitta G, Gennai S (2008). Single-center experience with the Talent LPS endograft in patients with at least 5 years of follow-up.. Journal of endovascular therapy: an official journal of the International Society of Endovascular Specialists.

[18] Torsello G, Osada N, Florek HJ, Horsch S, Kortmann H (2006). Long-term outcome after Talent endograft implantation for aneurysms of the abdominal aorta: a multicenter retrospective study.. Journal of vascular surgery: official publication, the Society for Vascular Surgery and International Society for Cardiovascular Surgery, North American Chapter.

[19] Seriki DM, Ashleigh RJ, Butterfield JS, England A, McCollum CN (2006). Midterm follow-up of a single-center experience of endovascular repair of abdominal aortic aneurysms with use of the Talent stent-graft.. Journal of vascular and interventional radiology: JVIR.

[20] Jones JE, Atkins MD, Brewster DC, Chung TK, Kwolek CJ (2007). Persistent type 2 endoleak after endovascular repair of abdominal aortic aneurysm is associated with adverse late outcomes.. Journal of vascular surgery: official publication, the Society for Vascular Surgery and International Society for Cardiovascular Surgery, North American Chapter.

[21] Parry DJ, Kessel DO, Robertson I, Denton L, Patel JV (2002). Type II endoleaks: predictable, preventable, and sometimes treatable?. Journal of vascular surgery: official publication, the Society for Vascular Surgery [and] International Society for Cardiovascular Surgery, North American Chapter.

[22] Nevala T, Biancari F, Manninen H, Aho PS, Matsi P (2010). Type II endoleak after endovascular repair of abdominal aortic aneurysm: effectiveness of embolization.. Cardiovascular and interventional radiology.

